# Supplementary material for: Activation of Alternative Bilirubin Clearance Pathways Partially Reduces Hyperbilirubinemia in a Mouse Model Lacking Functional Ugt1a1 Activity
Source: Int J Mol Sci. 2022 Sep 14;23(18):10703. doi: 10.3390/ijms231810703 (PMC9505366; doi:10.3390/ijms231810703)
Supplement: Supplementary file 1 [file ijms-23-10703-s001.zip › ijms-1841342-supplementary.pdf]

Supplementary Materials

Activation of alternative bilirubin clearance pathways partially reduces hyperbilirubinemia in a mouse model lacking functional Ugt1a1 activity

Bhaswati Banjeree [1], Joseph Olayemi Olajide [1,2], Giulia Bortolussi [1] and Andrés F. Muro [1,*]

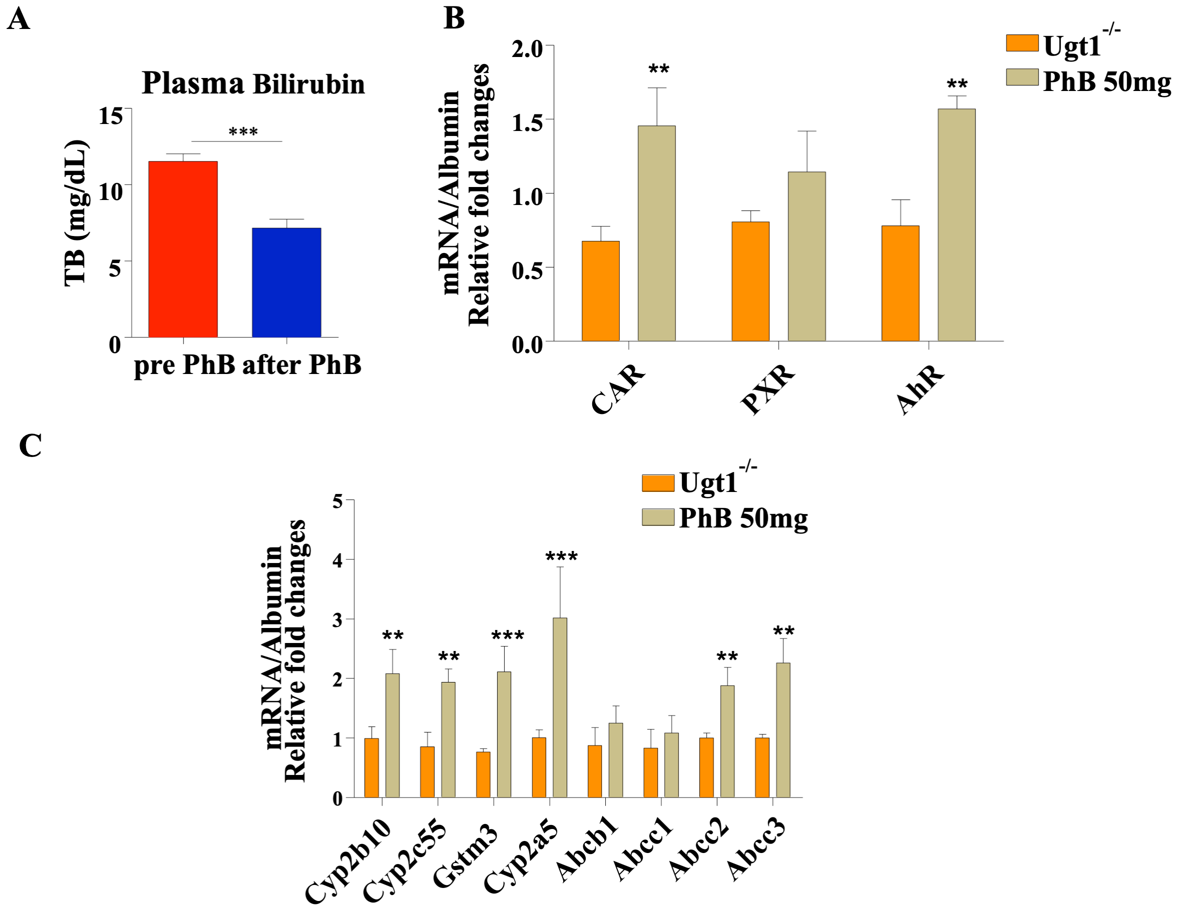

Figure S1: Phenobarbital (PhB) treatment decreases the plasma bilirubin level in adult Ugt1$^{-/-}$ animals

(A) Plasma total bilirubin level was measured before and after PhB treatment (n=3). Values represent mean ± SD (mg/dL). **(B)** qRT-PCR analysis of CAR, PXR and AhR in PBS treated (vehicle control) and PhB treated groups (n=3). Albumin was used as the internal control. Values represent mean ± SD. **(C)** QRT-PCR analysis of Cyp2b10, Cyp2c55, Gstm3, Cyp2a5, Abcb1, Abcc1, Abcc2 and Abcc3 in PBS treated (vehicle control) and PhB treated groups (n=3). Albumin was used as the internal control. Values represent mean ± SD, t-test was performed. ***p<0.001, **p<0.01 were interpreted as statistically significant.

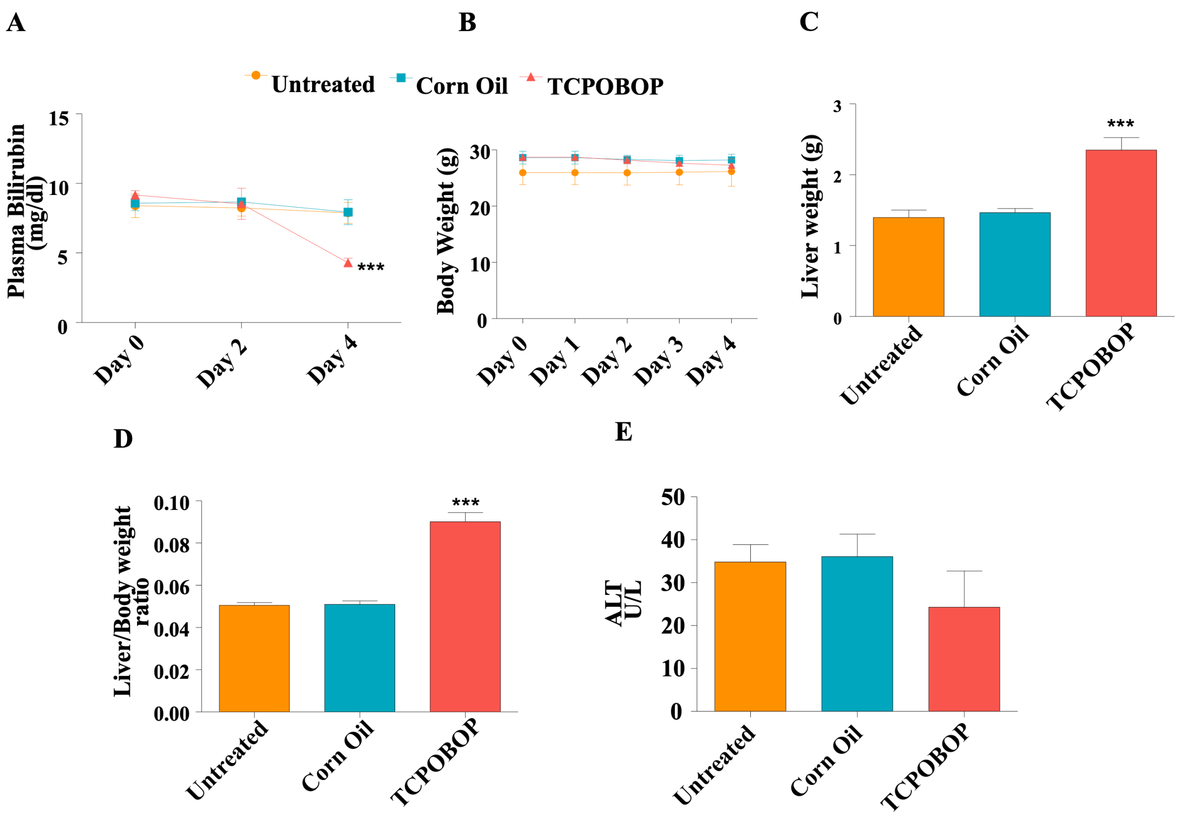

Figure S2: TCPOBOP treatment in adult Ugt1$^{-/-}$ animals

(A) Plasma total bilirubin level (mg/dL)in different treatment groups (n=6) at different time-points of the experiments. **(B)** Body weights (g) of animals in different treatment groups (n=6) at different time points of the experiment. **(C)** Liver weight (g) of animals in different treatment groups (n=6). Values represent mean ± SD. **(D)** Liver weight (g) and Body weight (g) ratio of animals in different treatment groups (n=6). Values represent mean ± SD. **(E)** Plasma ALT (U/L) level in the animals from different treatment groups (n=6). Values represent mean ± SD. One way ANOVA test was followed by Bonferroni's multiple comparison test. ***p<0.001 was interpreted as statistically significant. The treatment groups were: Untreated, Corn Oil (vehicle control) and TCPOBOP.

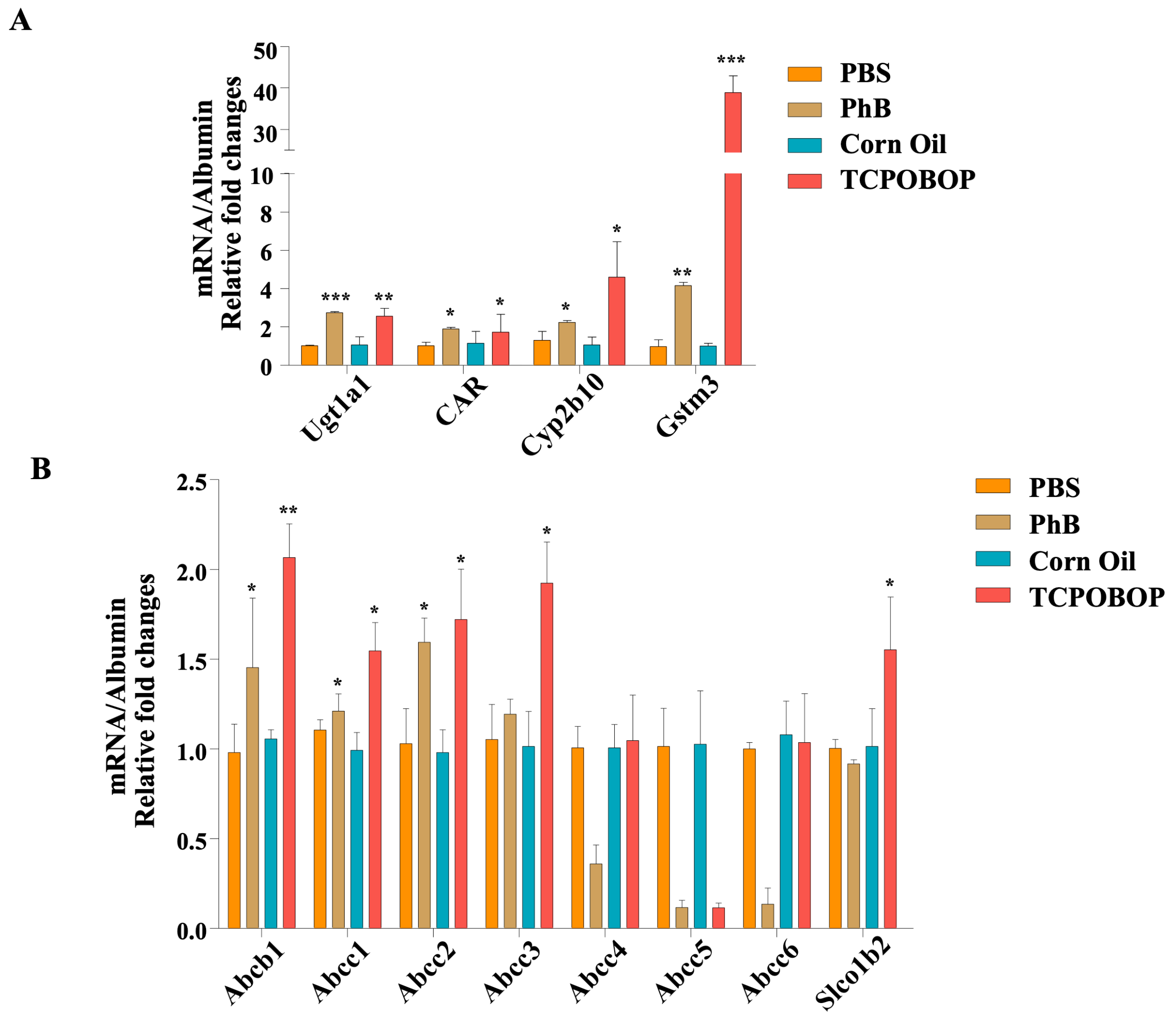

Figure S3: PhB and TCPOBOP treatment in adult Wild Type animals

(A) qRT-PCR analysis of Ugt1a1, CAR, Cyp2b10 and Gstm3. Albumin used as the internal control.
(B) qRT-PCR analysis of Abcb1, Abcc1, Abcc2, Abcc3, Abcc4, Abcc5, Abcc6, Slco1b2 in different treatment groups. Albumin was used as the internal control. Values represent mean ± SD. One way ANOVA test was followed by Bonferroni's multiple comparison test. ***$p<0.001$, **$p<0.01$, *$p<0.05$ were interpreted as statistically significant (n=3 for each treatment group).

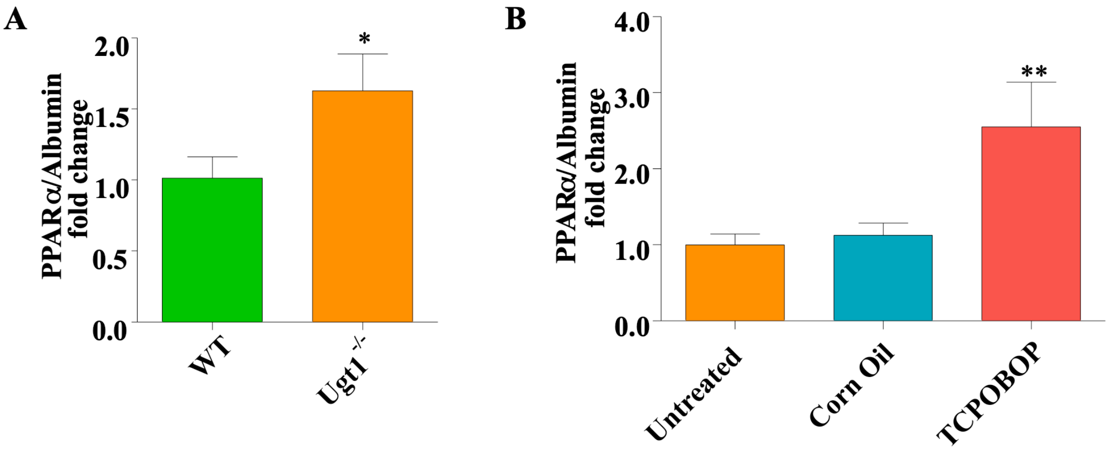

Figure S4: Modulation of PPARα expression in Ugt1$^{-/-}$animals upon TCPOBOP treatment.

(A) qRT-PCR analysis of PPARα in WT and Ugt1-/-animals. Albumin was used as the internal control (n=3 for each treatment group). Values represent mean ± SD. t-test was performed. *p<0.05 was interpreted as statistically significant. **(B)** qRT-PCR analysis of PPARα in different treatment groups. Albumin was used as the internal control (n=3 for each treatment group). The treatment groups were: Untreated, Corn Oil (vehicle control) and TCPOBOP. Values represent mean ± SD. One way ANOVA test was followed by Bonferroni's multiple comparison test. *p<0.05 , **p<0.01 was interpreted as statistically significant.

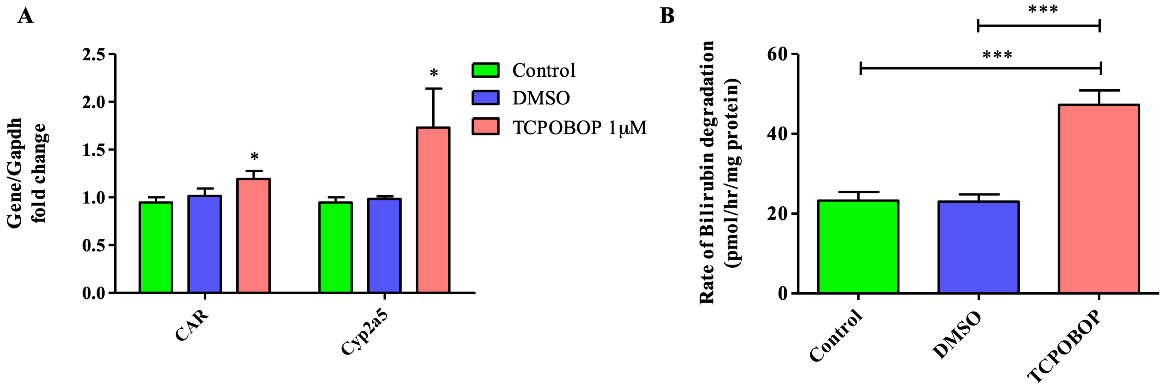

Figure S5: In-vitro treatment of TCPOBOP to Nmuli cells.

(A) qRT-PCR analysis of CAR and Cyp2a5. Gapdh was used as the internal control. *p<0.05 was interpreted as statistically significant. **(B)** Bilirubin disappearance assay using isolated microsomes from different treatment groups (Control, DMSO treated, TCPOBOP: 1 μM, 24 hr). Isolated microsomes were incubated with 10 μM bilirubin and the degradation rate was expressed as pmol/hr/mg protein. For our experiments ε= 72.692 cm^{-1} mM^{-1} was used. For the blank the difference in absorbance for the sample without NADPH was used. Values represent mean ± SD. One way ANOVA test was followed by Bonferroni's multiple comparison test. ***p<0.001, *p<0.05 were interpreted as statistically significant.
